# Estrogen and progesterone promote breast cancer cell proliferation by inducing cyclin G1 expression

**DOI:** 10.1590/1414-431X20175612

**Published:** 2018-03-01

**Authors:** J-M. Tian, B. Ran, C-L. Zhang, D-M. Yan, X-H. LI

**Affiliations:** 1 Department of Physiology, Southwest Medical University, Luzhou, China; 2 Department of Biochemistry, Southwest Medical University, Luzhou, China

**Keywords:** Breast cancer, Estrogen, Progesterone, Cyclin G1, Cell proliferation

## Abstract

Breast cancer is the most common cause of cancer among women in most countries (WHO). Ovarian hormone disorder is thought to be associated with breast tumorigenesis. The present study investigated the effects of estrogen and progesterone administration on cell proliferation and underlying mechanisms in breast cancer MCF-7 cells. It was found that a single administration of estradiol (E2) or progesterone increased MCF-7 cell viability in a dose-dependent manner and promoted cell cycle progression by increasing the percentage of cells in the G2/M phase. A combination of E2 and progesterone led to a stronger effect than single treatment. Moreover, cyclin G1 was up-regulated by E2 and/or progesterone in MCF-7 cells. After knockdown of cyclin G1 in MCF-7 cells using a specific shRNA, estradiol- and progesterone-mediated cell viability and clonogenic ability were significantly limited. Additionally, estradiol- and progesterone-promoted cell accumulation in the G2/M phase was reversed after knockdown of cyclin G1. These data indicated that estrogen and progesterone promoted breast cancer cell proliferation by inducing the expression of cyclin G1. Our data indicated that novel therapeutics against cyclin G1 are promising for the treatment of estrogen- and progesterone-mediated breast cancer progression.

## Introduction

Breast cancer is a serious health problem in females worldwide. The incidence and mortality rates of this disease are increasing rapidly. According to GLOBOCAN 2008, breast cancer is the most frequently diagnosed cancer and leading cause of cancer-related deaths among females, accounting for 458,000 of total cancer cases and 1,380,000 of deaths, making it the most common non-skin cancer in women (1,2). The etiology of breast cancer is very complex. Current knowledge considers this malignancy a multistep disease that involves the coordinated interaction of multiple genes and accumulation of multiple molecular and morphologic changes within a cell (3). Although these intracellular changes are influenced by the transcriptional control of key genes (4,5) or epigenetic modifications of histones (6,7), hormone disorders may be the most important determinants (8).

Epidemiological and experimental evidence have implicated estrogens as the most important risk factors in the etiology of breast cancer; estrogens are thought to function through hormone-related pathways (9). Moreover, endogenous estrogens are strongly associated with an increased risk of breast cancer in postmenopausal women (10), whereas the anti-estrogens raloxifene and tamoxifen reduce the incidence of breast cancer (11). Evidence from animal studies also supports that estrogens promote mammary tumors, and a decreased exposure to estrogens has an opposite effect (12). Thus, disorders of endogenous hormones, particularly estrogens, are great risk factors for human breast cancer.

However, the effects of estrogen alone do not fully account for breast cancer development. Other hormones such as progesterone may also be involved. Thus, the current study aimed to 1) investigate whether progesterone alone or in combination with estrogen affects breast cancer cell proliferation, and 2) explore the possible mechanisms underlying hormone-mediated breast cancer progression *in vitro*. Given that estradiol (E2) is the principal form of estrogen, breast cancer MCF-7 cells were treated with E2 to provoke the effects mediated by estrogen.

## Material and Methods

### Reagents and cell culture

E2 and progesterone were purchased from Sigma Co. (USA). A specific shRNA against cyclin G1 (shCyclin G1) was designed and synthesized by GenePharma (Shanghai, China). Scrambled shRNA was also synthesized as a negative control. Specific primary antibodies against cyclin G1 and glyceraldehyde-phosphate dehydrogenase (GAPDH) were commercially purchased from Santa Cruz Biotech. (USA). Breast cancer MCF-7 cells were obtained from American Type Culture Collection (ATCC, USA). Cells were maintained in RPMI 1640 (Gibco, USA) containing 10% fetal bovine serum (Gibco) at 37°C in humidified atmosphere of 5% CO_2_.

### Lentivirus-mediated RNA interference of cyclin G1

The small hairpin RNA (shRNA)-encoding oligonucleotides for cyclin G1 (shCyclin G1) were synthesized by Sangon (China). The lentivirus-delivered shCyclin G1 was packaged as previously described (13).

### Quantitative real-time PCR (qRT-PCR)

Total RNAs of cell lysates were extracted using Trizol reagent (Invitrogen, USA) 24 h post-infection and were immediately inversely transcribed into cDNA, using a PrimerScript RT reagent Kit (Takara, Japan) according to the manufacturer's instructions. The cDNAs were then subjected to quantitative PCR with primers chemically synthesized by Sangon Biotechnology (Shanghai, China). Primers sequences were as follows: cyclin G1, forward: 5′-TCTAAGCTTATGATAGAGGTACTGACAAC-3′, reverse: 5′-TTTGAATTCTGTAATAATCCAGTTAAGG-3′; GAPDH, forward: 5′-ACCACAGTCCATGCCATCAC-3′, reverse: 5′-TCCACCACCCTGTTGCTGTA-3′.

Real-time PCR was performed using SYBR Premix EX Tag (Takara) on an ABI 7900 thermal cycler (Applied Biosystems, USA). The PCR conditions were as follows: 95°C for 30 s, 40 cycles of 95°C for 5 s, and 60°C for 34 s. All experiments were performed in triplicate.

### Western blotting

MCF-7 cells were harvested 48 h after infection in each group. The total protein concentration was determined by the Bio-Rad DC protein Assay (Bio-Rad, USA). Equal amounts of protein (30 µg) were fractionated by 10% SDS-PAGE and transferred to polyvinylidene fluoride membranes. Thereafter, the membranes were blocked with 5% fat-free milk for 1 h and subjected to incubation with the primary antibodies overnight at 4°C. Membranes were then incubated with the corresponding secondary antibodies at room temperature for 1 h. The reactivity was developed by enhanced chemiluminescent autoradiography (ECL kit, Amersham, UK).

### Cell viability assay

A cell counting kit-8 (CCK-8, Japan) assay was conducted to assess cell viability under distinct treatments. Briefly, MCF-7 cells subjected to the indicated treatments were plated in 96-well plates at an initial density of 2×10^3^ per well. Cell numbers were evaluated for 5 consecutive days or on the first and fifth day after plating, based on the experimental design. Before each time point, an aliquot of 10 μL of CCK-8 reagent was added to each well and the cells were further incubated for 4 h at 37°C in a humidified incubator. The absorbance was measured at 450 nm, using a microplate reader (Molecular Devices, USA). The experiments were assayed in triplicate and repeated at least three times.

### Colony formation assay

MCF-7 cells were treated with E2 or progesterone either alone or in combination prior to testing. Briefly, MCF-7 cells under each treatment were seeded into 12-well plates (300 cells per well) to allow for continuous free growth. At the end of the 14th day, the cells were stained with crystal violet (Beyotime, China) and the number of colonies in each group was quantified. A colony was considered only when the number of assembled cells was greater than 50.

### Cell cycle analysis

MCF-7 cells in the logarithmic growth phase were harvested and randomly seeded into 6-well plates at an initial density of 5×10^6^ cells per well. After overnight culture, MCF-7 cells were treated as indicated. Cells were then harvested and 1 mL of propidium iodide stain was added to each sample. Thereafter, the percentage of cells in each cell cycle phase was analyzed by flow cytometry on a FACScan instrument (BD Biosciences, USA).

### Statistical analysis

Data are reported as means±SD. Student's *t*-test was performed to evaluate the statistical difference between groups. A P value of less than 0.05 was considered statistically significant.

## Results

### Estradiol or progesterone alone promoted MCF-7 cell viability

**Figure 1.**
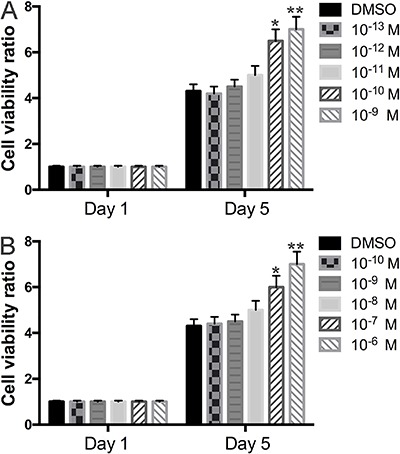
Effect of different concentrations of estradiol (*A*) or progesterone (*B*) alone in MCF-7 cell viability. DMSO was used as control. Data are reported as means±SD. *P<0.05; **P<0.01 *vs* DMSO group (control) (Student’s *t*-test).

Initially, we assessed the effects of E2 or progesterone on MCF-7 cell viability. To this end, MCF-7 cells were treated with E2 at final concentrations of 10^-13^, 10^-12^, 10^-11^, 10^-10^, and 10^-9^ M, and with progesterone at final concentrations of 10^-10^, 10^-9^, 10^-8^, 10^-7^, and 10^-6^ M. Cell viability was significantly increased on the fifth day of treatment with E2. Higher concentrations of E2 had even greater effects on MCF-7 cell viability (Figure 1A). Similarly, single treatment of MCF-7 cells with progesterone led to increased number of viable cells in a dose-dependent manner (Figure 1B). These data indicated that treatment of MCF-7 cells with either E2 or progesterone alone was sufficient for promoting cell viability *in vitro*.

### Combination of estradiol and progesterone had a greater effect on cell proliferation

**Figure 2.**
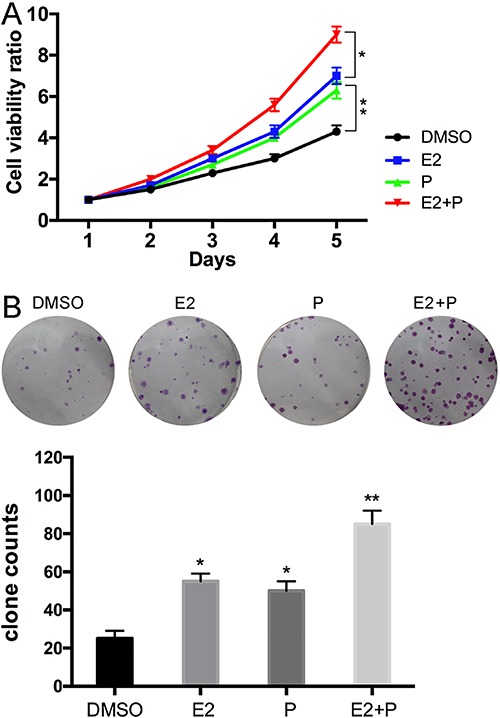
Combination of estradiol (E2, 10^-10^ M) and progesterone (P, 10^-8^ M) in cell proliferation effects. *A*, Cell viability in each group was monitored over five continuous days. *B*, Colony formation ability in each group of cells stained with crystal violet (upper panel) and counted (lower panel). Data are reported as means±SD. *P<0.05; **P<0.01 *vs* DMSO group (control) (Student’s *t*-test).

Next, we treated MCF-7 cells with E2 alone, progesterone alone, or a combination of both. In the cell viability assay, treatment of MCF-7 cells with either E2 or progesterone had a continuous viability promotion effect, although progesterone appeared to have a relatively lower ability to promote viability than estradiol. Notably, after combined treatments with E2 and progesterone, MCF-7 cells showed the highest viability among all groups (Figure 2A). Moreover, in the colony formation assay, a combination of E2 and progesterone resulted in greater colony numbers than single treatments in MCF-7 cells (Figure 2B). These data indicated that E2 combined with progesterone promoted higher proliferation than either single treatment.

### Estradiol and progesterone treatment promoted cell cycle progression

**Figure 3.**
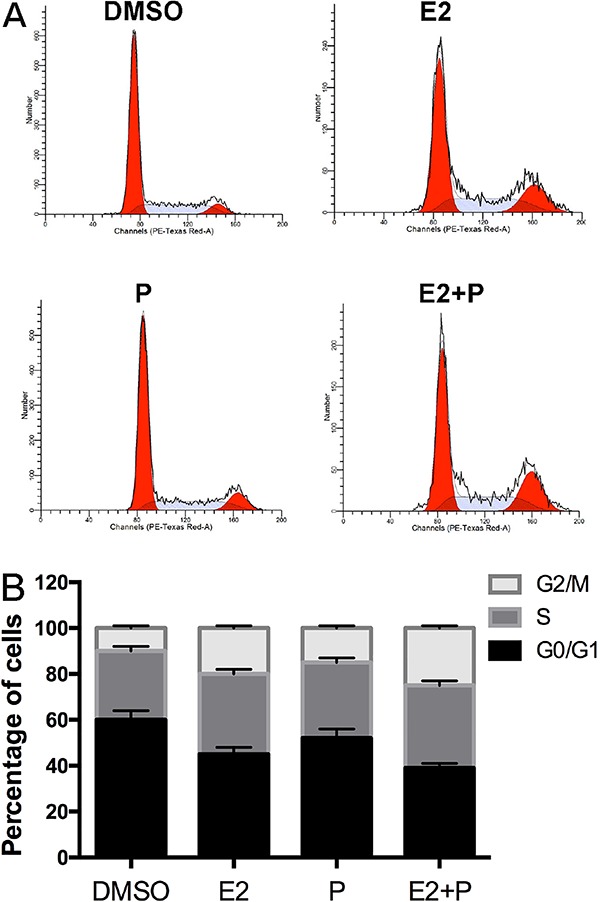
*A*, Effect of estradiol (E2) and progesterone (P) treatments on cell cycle progression. *B*, Cell accumulation in the G2/M phase of the cell cycle with each treatment. DMSO was used as control. Data are reported as means±SD.

Since the dysregulation of cell cycle progression is a hallmark of tumor growth (14), we assessed the percentage of cells in each cell cycle phase in MCF-7 cells after different treatments (Figure 3A). Our data showed that when MCF-7 cells were treated with either E2 or progesterone, the cell cycle distribution was disturbed and cells accumulated in the G2/M phase. Moreover, when the cells were treated with both E2 and progesterone, the percentage of cells in G2/M phase showed an even greater increase, whereas the G0/G1 population were significantly decreased (Figure 3B). These data indicated that cell cycle progression was promoted by estradiol and progesterone and the effect was greater with a combination of the two.

### Estradiol and progesterone treatments increased the expression of cyclin G1

**Figure 4.**
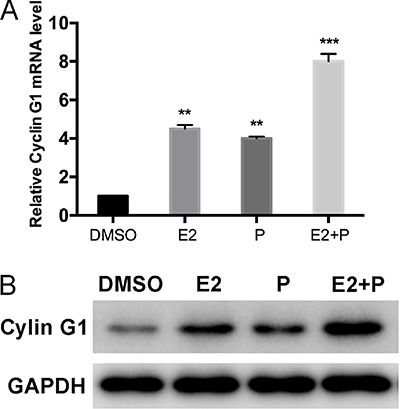
Effect of estradiol (E2) and progesterone (P) treatments on the mRNA expression of cyclin G1 (*A*), and on the protein level of cyclin G1 (*B*). Data are reported as means±SD. **P<0.01 *vs* DMSO; ***P<0.001 *vs* DMSO (Student’s *t*-test).

Cyclin G1 is a critical regulator of cell cycle progression. Notably, our data showed that treatment of MCF-7 cells with either E2 or progesterone increased the expression of cyclin G1 at both the mRNA (Figure 4A) and protein (Figure 4B) levels. More importantly, co-treatment with E2 and progesterone caused an even higher expression of cyclin G1. These data indicate that cyclin G1 was regulated by E2 and progesterone in MCF-7 cells.

### Knockdown of cyclin G1 reduced estradiol- and progesterone-mediated MCF-7 cell proliferation

**Figure 5.**
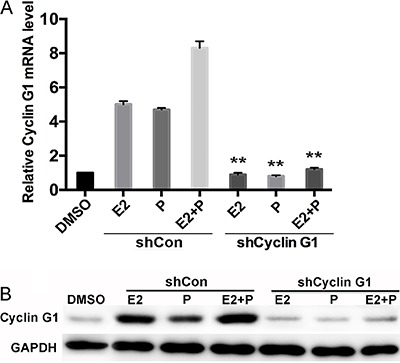
Synthesized shRNA against cyclin G1 (shCyclin G1) successfully depleted the expression of cyclin G1 in MCF-7 cells. *A*, Cyclin G1 mRNA expression was detected by q-PCR in the cells treated with estradiol (E2) and progesterone (P). *B*, The protein level of cyclin G1 was detected by western blot. Data are reported as means±SD. **P<0.01 *vs* shCon (scrambled shRNA as control) (Student’s *t*-test).

To assess whether cyclin G1 has a functional role in the E2- and progesterone-mediated MCF-7 cell proliferation, a specific shRNA targeting cyclin G1 (shCyclin G1) was used to deplete the expression of cyclin G1 in MCF-7 cells. As shown in Figure 5, E2 and progesterone treatment (either alone or combined) enhanced the expression of cyclin G1, whereas infection of MCF-7 cells with shCyclin G1 significantly decreased the mRNA level of cyclin G1 (Figure 5A). The protein level of cyclin G1 was also consistently decreased upon infection with shCyclin G1 (Figure 5B). These data indicated the high efficiency of synthesized shCyclin G1 in downregulating cyclin G1.

**Figure 6.**
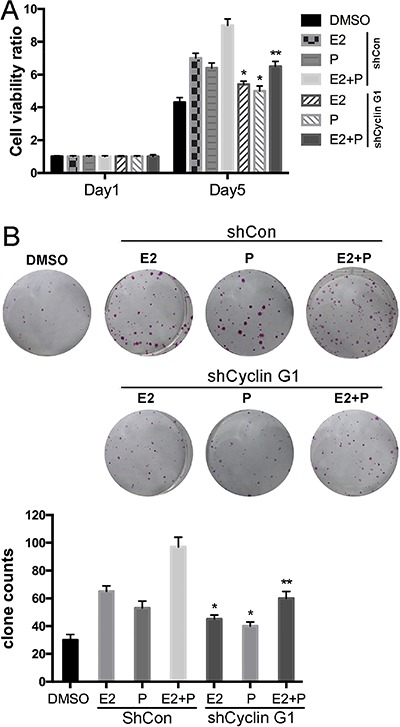
*A*, Knockdown of cyclin G1 (shCyclin G1) limits estradiol (E2)- and progesterone (P)-mediated MCF-7 cell proliferation. *B*, Knockdown of cyclin G1 decreased the colony number by up to 28% in single E2 treatment groups, 25.5% in single P treatment groups, and 40% in E2+P treatment groups (lower panel). Data are reported as means±SD. *P<0.05 and **P<0.01 *vs* shCon groups (Student's *t*-test).

Next, MCF-7 cells depleted or not of cyclin G1 (shCyclin G1 groups) were treated with the indicated hormones and subjected to cell viability determination and colony formation assay. The viability of cells depleted of cyclin G1 was significantly lower on the fifth day compared with that in the corresponding samples (Figure 6A). Colony counting revealed that knockdown of cyclin G1 decreased the colony number by up to 28% in E2 alone treatment groups, 25.5% in progesterone alone treatment groups, and 40% in E2 plus progesterone treatment groups (Figure 6B). These data strongly indicate that knockdown of cyclin G1 limited E2- and progesterone-mediated cell proliferation *in vitro*.

### Knockdown of cyclin G1 limited estradiol- and progesterone-mediated cell accumulation in G2/M phase in MCF-7 cells

**Figure 7.**
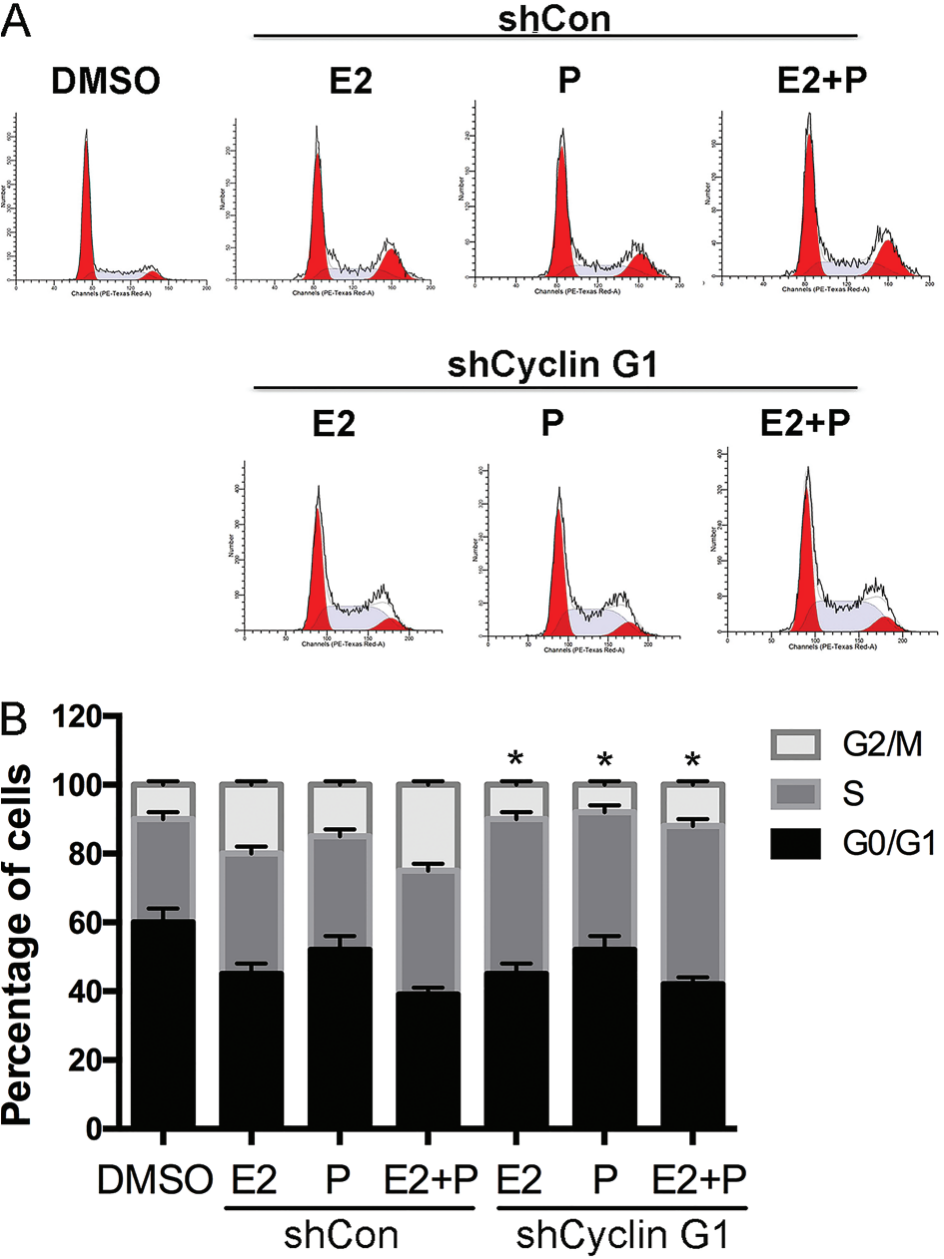
Knockdown of cyclin G1 (shCyclin G1) limits estradiol (E2)- and progesterone (P)-mediated cell accumulation in G2/M phase in MCF-7 cells. *A*, Cell cycle analysis. *B*, Percentage of cells in cell cycle phases with each treatment. Data are reported as means±SD. *P<0.05 *vs* shCon (Student’s *t*-test).

Next, we analyzed cell cycle progression in MCF-7 cells infected with or without shCyclin G1 (Figure 7A). Single or combined administration of E2 and progesterone caused cells to accumulate in the G2/M phase, which was consistent with the results of the cell cycle analysis described in Figure 3. More importantly, the percentage of cells in the G2/M phase was significantly decreased in the cyclin G1-depleted group versus the hormone-treated counterparts that were not depleted of cyclin G1. In contrast, the percentages of cells in the S phase were increased in cyclin G1-depleted groups compared to that in their control counterparts (Figure 7B). These observations indicated that knockdown of cyclin G1 reduced E2- and progesterone-mediated cell accumulation in the G2/M phase.

## Discussion

Previous studies showed that continuous hormone replacement treatment with estrogen plus progesterone is linked to a reduced risk of endometrial cancer (15,16), but associated with an increased risk of developing breast cancer (17). These data indicate that estrogen and progesterone are involved in the development of breast cancer.

The present study investigated the effects of estrogen plus progesterone on breast cancer MCF-7 cell proliferation. As ligands of the receptors, estrogen and progesterone are thought to have functional roles in MCF-7 cell proliferation. The results of this study showed that administration of estrogen (mainly estradiol) or progesterone alone was sufficient to promote MCF-7 cell proliferation and clonogenic abilities. After a 5-day treatment, E2 and progesterone increased MCF-7 cell proliferation in a dose-dependent manner. Furthermore, E2 and progesterone promoted cell cycle progression by accumulating large number of cells in G2/M phase. Since dysregulated cell cycle progression is a hallmark of tumorigenesis (14,18–20), the cell cycle analysis results support our hypothesis that estrogen and progesterone promote MCF-7 cell proliferation. Furthermore, combined treatment of MCF-7 cells with E2 and progesterone caused even stronger effects on cell proliferation, indicating that progesterone can promote MCF-7 cell proliferation on its own (21), and enhance estrogen-mediated breast cancer cell proliferation. In fact, progesterone has been proposed to augment the effects of estrogen on breast cancer development (9). Therefore, our data indicate that progesterone and estrogen had a synergistic role in promoting tumor growth in MCF-7 cells.

One novel aspect of this study is that cyclin G1 was found to be a critical target gene that mediated estradiol- and progesterone-induced breast cancer cell proliferation. Cyclin G is a member of the cyclin family and contains a well-conserved cyclin box (22). Cyclins function by regulating the activities of cyclin-dependent kinases and are thereby involved in cell cycle regulation (14). Two members, cyclin G1 and cyclin G2, have been identified, of which cyclin G1 is a negative regulator of the tumor suppressor gene p53 (23). The negative regulation of p53 indicates that cyclin G1 promotes tumor growth. However, unlike other cyclins, cyclin G1 has two-sided effects on cell growth, depending on the cell type (24). For example, cyclin G1 is known to exert negative control of cell proliferation in endometrial carcinoma (24) in a progesterone-dependent manner (25). A deficiency in progesterone and its receptors is an important cause of decreased expression of cyclin G1 in endometrial carcinoma (25). In contrast, in hepatic tumors (26) and cervical carcinoma (27), overexpression of cyclin G1 has been shown to promote cell growth, which contradicts the results for endometrial carcinoma. These conflicting results indicate that cyclin G1 has a dual role in human tumorigenesis. In this study, we identified that cyclin G1 was under positive control by E2 and progesterone. Both E2 and progesterone promoted the expression of cyclin G1 in MCF-7 cells, which is consistent with a previous report (25). Functionally, knockdown of cyclin G1 blunted estradiol- and progesterone-mediated MCF-7 cell proliferation by 28 and 25.5%, respectively, as well as disrupted estrogen- and progesterone-mediated cell cycle progression in MCF-7 cells. These data indicate that in breast cancer, cyclin G1 is a positive regulator of cell proliferation despite its dual role in other cancer types. In contrast, our data suggest that targets against cyclin G1 are promising therapeutics for the treatment of breast cancer.

In summary, we found that E2 plus progesterone exerted greater detrimental effects on the risk of breast cancer than either E2 or progesterone alone. The increased proliferation of breast cancer cells was achieved by inducing the expression of cyclin G1. Therefore, therapeutics against cyclin G1 might prove to be promising for the treatment of breast cancer.
